# Similar improvements in cognitive inhibitory control following low-intensity resistance exercise with slow movement and tonic force generation and high-intensity resistance exercise in healthy young adults: a preliminary study

**DOI:** 10.1186/s12576-021-00806-0

**Published:** 2021-07-17

**Authors:** Kento Dora, Tadashi Suga, Keigo Tomoo, Takeshi Sugimoto, Ernest Mok, Hayato Tsukamoto, Shingo Takada, Takeshi Hashimoto, Tadao Isaka

**Affiliations:** 1 Faculty of Sport and Health Science, Ritsumeikan University, 1-1-1 Nojihigashi, Kusatsu, Shiga 525-8577 Japan; 2 Faculty of Lifelong Sport, Department of Sports Education, Hokusho University, Ebetsu, Hokkaido Japan

**Keywords:** Cognitive function, Brain health, Lactate, Blood pressure, Electromyographic activity, Perceived exertion

## Abstract

This study compared the effects of low-intensity resistance exercise with slow movement and tonic force generation (ST-LRE) and high-intensity resistance exercise (HRE) on post-exercise improvements in cognitive inhibitory control (IC). Sixteen young males completed ST-LRE and HRE sessions in a crossover design. Bilateral knee extensor ST-LRE and HRE (8 repetitions/set, 6 sets) were performed with 50% of one-repetition maximum with slow contractile speed and 80% of one-repetition maximum with normal contractile speed, respectively. The IC was assessed using the color–word Stroop task at six time points: baseline, pre-exercise, immediate post-exercise, and every 10 min during the 30-min post-exercise recovery period. The blood lactate response throughout the experimental session did not differ between ST-LRE and HRE (condition × time interaction *P* = 0.396: e.g., mean ± standard error of the mean; 8.1 ± 0.5 vs. 8.1 ± 0.5 mM, respectively, immediately after exercise, *P* = 0.983, *d* = 0.00). Large-sized decreases in the reverse-Stroop interference scores, which represent improved IC, compared to those before exercise (i.e., baseline and pre-exercise) were observed throughout the 30 min post-exercise recovery period for both ST-LRE and HRE (decreasing rate ≥ 38.8 and 41.4%, respectively, all *d*s ≥ 0.95). The degree of post-exercise IC improvements was similar between the two protocols (condition × time interaction *P* = 0.998). These findings suggest that despite the application of a lower exercise load, ST-LRE improves post-exercise IC similarly to HRE, which may be due to the equivalent blood lactate response between the two protocols, in healthy young adults.

## Introduction

Skeletal muscle weakness, as seen in decreased muscle mass and strength, is a prominent factor that indicates poor prognosis in older individuals and patients with chronic diseases [1]. Many people with skeletal muscle weakness also present decreased cognitive function [2], which is also a poor prognostic factor [3]. Because a complication of skeletal muscle weakness and decreased cognitive functions additively exacerbates physical inactivity [4], resolution of this public health problem worldwide is now urgent.

Resistance exercise is the most beneficial strategy for increasing skeletal muscle size and strength [5, 6]. Additionally, long-term intervention of resistance exercise improves cognitive function in healthy young and older individuals [7, 8]. Furthermore, long-term resistance exercise is effective in improving cognitive function in patients with chronic diseases, including cognitive impairment (e.g., mild cognitive impairment) [8]. Therefore, resistance exercise has recently been recognized as an effective strategy for enhancing skeletal muscle and cognitive health in various populations.

Cognitive function involves various capabilities, including executive function. The executive function is an advanced cognitive function that is primarily characterized by inhibition, shifting, updating, and other cognitive subcomponents [9]. Inhibitory control (IC) is a specific executive function that is defined as the suppression of behavior in response to either internal or external stimuli [10]. It is necessary for preventing the implementation of an unrequired action during cognitive processing [11]. Therefore, IC is important for all cognitive processes [12].

Previous studies have reported that an acute bout of whole-body resistance exercise involving multiple events (e.g., combinations of leg press, bench press, and lat pulldown, etc.) improves post-exercise IC in older individuals and patients with chronic diseases (e.g., mild cognitive impairment), as well as in healthy young individuals [e.g., [13–15]]. However, whole-body resistance exercise recruits a large number of muscle groups during the exercise session. A similar muscle recruitment pattern is also observed in aerobic exercise (e.g., cycling), because it is performed with movements of multiple joints (e.g., the ankle, knee, and hip joints). In contrast, localized resistance exercise (e.g., only knee extension), which is performed by a single-joint movement, recruits a limited number of muscle groups. Therefore, compared to whole-body resistance exercise, localized resistance exercise may help enhance our understanding of the underlying effects of resistance exercise-induced specific muscle stimulation on post-exercise cognitive function.

We have previously reported that localized resistance exercise, which performed with knee extension, improves post-exercise IC in healthy young adults [16, 17]. Of these studies, we found a greater immediate post-exercise IC improvement following high-intensity resistance exercise (HRE) than following low-intensity resistance exercise (LRE) [17]. This suggests that conventional LRE may be inadequate for improving IC. However, LRE is feasible for many populations [5, 6], because lower loads on physical and physiological systems (e.g., cardiovascular and musculoskeletal systems) compared with HRE. Therefore, it would be useful to identify effective strategies for enhancing LRE-induced IC improvement.

Previous studies have reported that long-term intervention of LRE with slow movement and tonic force generation (ST-LRE) increases skeletal muscle size and strength effectively in healthy young and older individuals [18–22]. Furthermore, Takenami et al. [23] reported that long-term ST-LRE was effective to increasing these muscle size and strength adaptations in patients with type 2 diabetes, which is a well-known risk of various cognitive diseases (e.g., mild cognitive impairment and Alzheimer’s disease) [24]. Additionally, Tanimoto et al. [19] reported that blood pressure response and electromyographic (EMG) activity during acute bout of the localized knee extensor resistance exercise were lower for ST-LRE than for HRE in healthy young adults. Their findings suggest that ST-LRE may have beneficial effects for mitigating the loads on cardiovascular and musculoskeletal systems compared with HRE. Therefore, despite the application of lower exercise loads, ST-LRE is an effective strategy in increasing skeletal muscle size and strength in various populations.

During exercise, lactate acts as an important energy substrate, instead of glucose, in the human brain [24]. Indeed, we have previously demonstrated that the difference in the increase of circulating lactate levels between the first and second bouts during repeated high-intensity interval exercise sessions is associated with the difference in the degree of post-exercise IC improvements [28, 30], potentially due to increasing cerebral lactate metabolism [28]. Considering these findings, the degree of the post-exercise IC improvements can be at least partially explained by the increase in blood lactate levels when high-intensity exercise is performed.

Tanimoto et al. [19] reported that the increase in blood lactate levels induced by an acute bout of knee extensor resistance exercise was similar between ST-LRE and HRE in healthy young adults. Hence, we hypothesized that ST-LRE would improve post-exercise IC similarly to HRE, with an equivalent blood lactate response between the two protocols. To test this hypothesis, in this study, we recruited healthy young adults in a preliminary safety study in order to identify whether ST-LRE would be an effective protocol for improving cognitive function in various populations. Therefore, the purpose of this study was to compare the effects of knee extensor ST-LRE and HRE on post-exercise IC in healthy young adults.

## Methods

### Subjects

Sixteen healthy, young males (mean ± standard error of the mean [SEM]: age: 21.9 ± 0.4 years, body height: 173.1 ± 1.1 cm, body weight: 62.6 ± 3.0 kg) participated in this study. Prior to this study, we calculated the required sample size utilizing an effect size of 0.31, an α-level of 0.05, and a β-level of 0.2 (80% power), based on the data (i.e., the reverse-Stroop interference score) of our previous study [30]. The calculated necessary number of subjects was 14; therefore, the number of subjects recruited in this study was sufficient for ensuring statistical power and sensitivity. The subjects were recreationally active and participated in physical exercise (e.g., resistance exercise and/or aerobic exercise) for 2–4 h per week. The subjects provided written informed consent upon having the experimental procedures and potential risks described to them. The color–word Stroop task (CWST) was performed using the subject’s right hand. All subjects were considered right-hand dominant, which was ascertained by asking each subject which hand they preferred to use for writing. Furthermore, they were free of any known neurological, cardiovascular, and pulmonary disorders, as well as free from color-blindness and abnormal vision. This study was approved by the Ethics Committee of Ritsumeikan University and conducted according to the Declaration of Helsinki.

### Experimental design

**Fig. 1:**
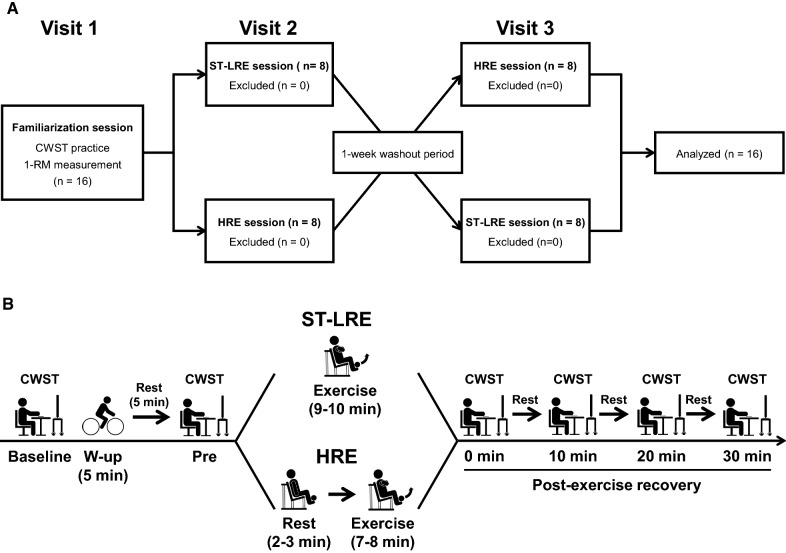
Experimental procedures of this study. Panel **A** shows the consort flow diagram for three visits of this study. In the familiarization day, subjects performed the color–word Stroop task (CWST) practice and one-repetition maximum (1-RM) measurement. In the two experimental days, subjects completed low-intensity resistance exercise with slow movement and tonic force generation (ST-LRE) and high-intensity resistance exercise (HRE) sessions in a crossover design with a randomized and counterbalanced order. Panel **B** shows experimental procedure during the two experimental days. The exercise loads for ST-LRE and HRE were set at 50% and 80% of 1-RM, respectively. The bilateral knee extension exercise for both ST-LRE and HRE was programmed for six sets with eight repetitions per set. The CWST was administered at baseline, before exercise, immediately after exercise, and every 10 min during the 30-min post-exercise recovery period

Experimental procedures of this study are presented in Fig. 1. On the day of familiarization visit, subjects practiced the three types of the CWST for each a minimum of 10 times until they achieved consistent scores, as in our previous studies [16, 17, 28, 30–37]. During the familiarization day, the subjects also completed measurement of one-repetition maximum (1-RM) of the bilateral knee extension, which was used to calculate exercise loads for ST-LRE and HRE.

On the two experiment days, the subjects visited after fasting overnight (i.e., abstinence from food for 12 h) and avoiding strenuous physical activity for 24 h prior to the experiment, as in our previous studies [16, 17, 28, 30–37]. The subjects also abstained from caffeine and alcohol for 12 h prior to the experiment and were not taking any medications that would affect their cognitive performance. Before the start of the experiment session, the subjects ingested approximately 100–200 ml of water that appears not to interfere with the experiment session. Thereafter, the subjects did not ingest water until the experiment session is completed, because dehydration during localized resistance exercise (i.e., shorter exercise duration) is generally expected to be low.

In the experiment session, the subjects first practiced the three CWST types for each a minimum of five times before experimental session to minimize the learning effect, as in our previous studies [16, 17, 28, 30–37]. Then, the subjects rested for 5 min before undergoing physiological and psychological parameter measurements. After these baseline data were collected, the subjects performed the baseline CWST.

Next, the subjects performed a warm-up exercise at 50 W for 5 min using a bicycle ergometer (Life Fitness; Schiller Park, IL, USA), as in our previous studies [16, 17]. Our previous studies have confirmed that duration and intensity of the warm-up exercise do not result in positive effect on IC [16, 17]. After the warm-up exercise, the subjects rested for 5 min and then performed the pre-exercise CWST to confirm that the warm-up exercise did not affect IC.

Subsequently, the subjects completed either ST-LRE or HRE. The CWST was performed again immediately after the completion of the exercise session and then repeated three times at 10-min intervals during the 30-min post-exercise recovery period to evaluate the sustainable effects of post-exercise IC improvements, similar to our previous studies [16, 28, 30, 32–37].

Cardiovascular parameters (i.e., heart rate [HR] and blood pressure), three quadriceps femoris EMG activities, and rating of perceived exertion (RPE) during the exercise session were measured in every set to assess the levels of physiological, neuromuscular, and perceptual responses. Fingertip blood samples were collected immediately before all six CWSTs to assess the effects of metabolic conditions on IC. The Felt Arousal Scale (FAS) and Visual Analogue Scale (VAS) were measured immediately after all CWSTs to assess the effects of psychological conditions on IC.

### Experimental protocols

All subjects completed both ST-LRE and HRE in a randomized and counterbalanced order (see Fig. 1A). The ST-LRE and HRE were set at 50% and 80% of 1-RM, respectively, according to previous studies [19, 20, 22]. Both protocols were programmed with bilateral knee extension for six sets with eight repetitions per set using a leg extension machine (Life Fitness; Schiller Park, IL, USA). The ST-LRE and HRE were performed with slow (3-s concentric, 3-s eccentric, and 1-s isometric actions with no rest between each repetition) and normal contractile speeds (1-s concentric, 1-s eccentric, and 1-s rest between each repetition), respectively. Rest intervals between sets for both protocols lasted 1 min. These resistance exercise variables were the basis of the methods employed in previous studies [19–23]. The two experimental sessions were performed at approximately the same time (± 1 h) in the morning, as separated by 1 week.

#### 1-RM

On the familiarization visit, subjects’ 1-RM was obtained by a successful concentric contraction of bilateral knee extension to calculate exercise loads for ST-LRE and HRE, as previously described [16, 17, 38]. The bilateral knee extensor 1-RM in all subjects was 116 ± 6 kg [mean ± SEM]. The loads used in ST-LRE and HRE were 58 ± 3 and 93 ± 5 kg, respectively.

#### HR

HR was measured continuously via telemetry (RS400; Polar Electro Japan, Tokyo, Japan). During exercise session, peak HR was corrected every set, and the mean value of all six sets was calculated for analysis of this study.

### Blood pressure

Systolic blood pressure (SBP) and diastolic blood pressure (DBP) were measured using a mercury manometer (FC-110ST; Focal, Chiba, Japan). During exercise session, both blood pressure variables were corrected every set, and the mean value of all six sets for each was calculated for analysis of this study.

### Quadriceps femoris EMG

Prior to the application of electrodes, subject’s skin was shaved, abraded, and cleaned with alcohol to minimize skin impedance. Surface EMG electrodes were placed along the longitudinal axis of the vastus lateralis (VL), vastus medialis (VM), and rectus femoris (RF) in the right leg based on the European Recommendations for surface electromyography [40]. The electrode placement on the VL was at approximately two-thirds of the distance between the anterior superior iliac spine and the lateral aspect of the patella. The electrode placement on the VM was at 20% of the distance from the medial joint line of the knee to the anterior superior iliac spine. The electrode placement on the RF was at 50% of the distance from the anterior superior iliac spine to the superior pole of the patella. The EMG signals for each muscle were amplified 1000 times, band-pass filtered between 10 and 500 Hz, and sampled at 1000 Hz (MQ-Air; Kissei Comtech, Nagano, Japan).

To determine the magnitude of the quadriceps femoris muscle activity, the EMG activities of the three muscles during exercise were quantified as the integration of the rectified EMG (iEMG). The peak iEMG over 1 s during each exercise repetition was normalized to that (mean value over 1 s) during a trial that recorded the highest iEMGs obtained during two trials of the knee extension maximal voluntary contraction, which was measured after the 30-min post-exercise recovery period. The peak iEMGs of each muscle were calculated from all eight repetitions per every set. The mean values of all six sets for each were used for the analysis of this study.

In a preliminary experiment, we examined the reliability of the three quadriceps femoris muscle activities (i.e., peak iEMGs) during the knee extensor maximal voluntary contraction on two separate days in 8 healthy young males. The coefficients of variation for the 2 days were 4.8 ± 1.3% [mean ± SEM] for the VL, 4.6 ± 1.8% for the VM, and 4.2 ± 0.8% for the RF. The intraclass correlation coefficients for the 2 days were 0.947 for the VL, 0.988 for the VM, and 0.982 for the RF, which can be considered as excellent reliability [41].

#### RPE

The Borg’s RPE scale was measured to assess the perceived exertion expended during exercise, which ranges from 6 (no exertion) to 20 (maximal exertion) [39]. During exercise session, RPE was corrected every set, and the mean value of all six sets was calculated for analysis of this study.

### Blood metabolites

Blood glucose and lactate levels were measured using a glucose (Medisafe FIT Blood Glucose Meter; Terumo, Tokyo, Japan) and lactate analyzer (Lactate Pro 2; Arkray, Kyoto, Japan), respectively.

### Psychological parameters

FAS is a 6-point, single-item scale ranging from 1 (low arousal) to 6 (high arousal) [42]. VAS consisted of questions of three psychological types that assess mental fatigue, the ability to concentrate, and motivation. Each VAS was labeled from 0 mm (i.e., not at all) to 100 mm (i.e., extremely). Subjects drew lines to indicate their response.

#### CWST

All CWSTs throughout experimental session were performed in a quiet room with as little noise as possible. This room was set at moderate temperature of approximately 24 ºC, which is a temperature that does not affect cognitive performance [43]. The method of the CWST has been described in our previous study [8, 9, 15–23]. In brief, stimuli words were four color names (i.e., RED, YELLOW, GREEN and BLUE), and they were presented on a 98-in. display. The three types of the CWST consisted of two color text tasks (i.e., congruent and incongruent tasks) and one control black text task (i.e., neutral task). One trial of each type of the CWST consisted of 24 stimulus words. The three CWST types were repeated for each three trials. All CWSTs throughout experimental session were measured by the same examiner who was familiar with this procedure.

The reaction time and response accuracy for each trial (i.e., a total of nine trials) were collected. Then, these values of the three trials for the three CWST types were averaged and the mean values were used for analysis of this study. The IC was assessed using the reverse-Stroop interference score, which is defined as the difference between reaction times of the neutral and incongruent tasks. The reason for using the reverse-Stroop interference score to determine IC has been described in our previous study [33, 34]. The reverse-Stroop interference score was calculated as [(reaction time of incongruent task – reaction time of neutral task)/reaction time of neutral task × 100] [44].

### Statistical analysis

All data are expressed as mean ± SEM. Mean values of cardiovascular parameters (i.e., HR, SBP, and DBP) and quadriceps EMG activities between ST-LRE and HRE were compared using a paired Student’s *t*-test. Changes in measured variables throughout experimental session between the two protocols were analyzed using two-way (condition × time) repeated-measures analysis of variance. If the sphericity assumption was not met, Greenhouse–Geisser corrections were used. Specific differences were identified with a Bonferroni post hoc test. The statistical significance level was defined at *P* < 0.05. The Cohen’s *d* effect size using the pooled SD was calculated to determine the magnitude of a difference between two values. The strength of this effect size can be interpreted as small (0.20 ≤ *d* < 0.50), medium (0.50 ≤ *d* < 0.80) and large (0.80 ≤ *d*) [45]. Partial eta-squared (*η*_p_^2^) was determined as the effect size for main effects of condition and time or interaction effect. All statistical analyses were conducted using IBM SPSS software (Ver. 19.0, IBM Corp, NY, USA).

## Results

### Cardiovascular variables before exercise

HR and blood pressure variables before exercise (i.e., pre-exercise) did not differ significantly between ST-LRE and HRE (HR: 70.0 ± 1.9 vs. 69.3 ± 1.5 bpm, SBP: 115.4 ± 1.7 vs. 112.9 ± 1.9 mmHg, DBP: 68.4 ± 0.7 vs. 69.3 ± 1.1 mmHg, respectively, all *P*s > 0.05, *d* = 0.10 to 0.35).

### Measured variables during exercise

Mean values of cardiovascular responses and quadriceps femoris EMG activities during ST-LRE and HRE are shown in Table 1. Mean HR during exercise did not differ significantly between ST-LRE and HRE. Although there was no significant difference for mean DBP during exercise between the two protocols, mean SBP during exercise was significantly lower for ST-LRE than for HRE (*P* < 0.001, *d* = 0.69). Additionally, peak iEMGs of all three quadriceps femoris muscles during exercise were significantly lower for ST-LRE than for HRE (all *P*s < 0.01, *d* = 0.96 to 1.60). Mean RPE did not differ significantly between the two protocols (*P* = 0.379, *d* = 0.22).

**Table 1:** Mean values of cardiovascular parameters, quadriceps femoris electromyographic activities, and rating of perceived exertion during low-intensity resistance exercise with slow movement and tonic force generation (ST-LRE) and high-intensity resistance exercise (HRE)

	ST-LRE	HRE
Heart rate, bpm	116.1 ± 2.6	118.4 ± 2.6
Systolic blood pressure, mmHg	131.7 ± 1.7	138.3 ± 2.9*
Diastolic blood pressure, mmHg	66.7 ± 1.6	64.6 ± 1.4
Peak iEMG		
Vastus lateralis, % of MVC	83.7 ± 4.3	104.2 ± 5.0*
Vastus medialis, % of MVC	79.3 ± 6.3	102.4 ± 5.7*
Rectus femoris, % of MVC	65.1 ± 4.5	102.9 ± 7.0*
Rating of perceived exertion	14.6 ± 0.4	14.4 ± 0.2

Values are presented as mean ± standard error of the mean (SEM). Each variable was calculated as mean value of all six sets. *iEMG* integrated electromyography, *MVC* maximal voluntary contraction. *Significant difference between ST-LRE and HRE

### Changes in blood metabolites throughout experimental session

**Fig. 2:**
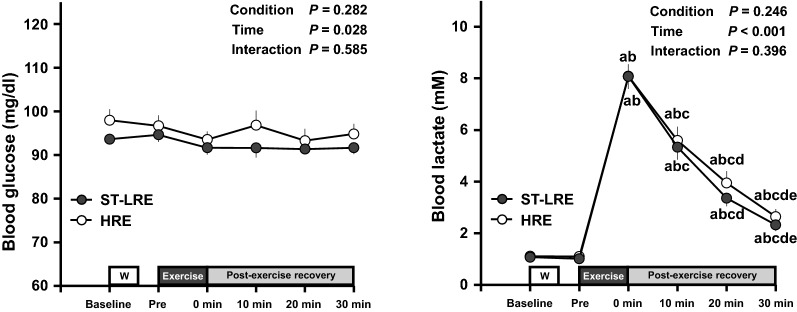
Changes in blood glucose and lactate levels throughout ST-LRE and HRE sessions. Values are presented as mean ± standard error of the mean (SEM). ^a^*P* < 0.001 vs. Baseline. ^b^*P* < 0.001 vs. before exercise (i.e., Pre). ^c^*P* < 0.001 vs. immediately after exercise (i.e., 0 min post-exercise recovery period). ^d^*P* < 0.001 vs. 10 min post-exercise recovery period. ^e^*P* < 0.001 vs. 20-min post-exercise recovery period

Changes in blood metabolites throughout ST-LRE and HRE sessions are presented in Fig. 2. Blood glucose analysis revealed a significant main effect for time (*F*_(5,75)_ = 2.68, *P* = 0.028, *η*_p_^2^ = 0.15). However, there was no significant difference in blood glucose between ST-LRE and HRE throughout experimental sessions. Blood lactate analysis revealed a significant main effect for time (*F*_(1.44, 21.59)_ = 158.05, *P* < 0.001, *η*_p_^2^ = 0.91). Blood lactate significantly increased immediately after ST-LRE and HRE compared with that before each exercise (all *P*s < 0.001, *d* = 5.08 to 5.29 vs. baseline and pre-exercise for both protocols). The increased blood lactate remained significant until the 30-min post-exercise recovery period for both protocols (all *P*s < 0.01, *d* = 1.79 to 1.90 vs. baseline and pre-exercise for both protocols).

### Changes in the CWST-measured IC throughout experimental session

Changes in reaction times and response accuracies on three types of the CWST throughout ST-LRE and HRE sessions are summarized in Table 2. Reaction time analyses for all three CWST types revealed significant main effects for time (*F*_(5,75)_ = 3.55, *P* = 0.006, *η*_*p*_^*2*^ = 0.19 for congruent task; *F*_(5,75)_ = 5.31, *P* < 0.001, *η*_p_^2^ = 0.26 for neutral task; *F*_(5,75)_ = 25.11, *P* < 0.001, *η*_p_^2^ = 0.63 for incongruent task). Although the congruent reaction time did not differ significantly among all time points throughout both experimental sessions, the neutral and incongruent reaction times significantly decreased immediately after ST-LRE and HRE compared with that before each exercise (all *P*s < 0.05, *d* = 0.31 to 0.57 vs. baseline and/or pre-exercise for both protocols). The decreased incongruent reaction time following ST-LRE and HRE remained significant until the 20 min (both *P*s < 0.01, *d* = 0.50 and 0.56 vs. baseline and pre-exercise, respectively) and 30 min (*P* = 0.002, *d* = 0.45 vs. baseline), respectively, of post-exercise recovery periods. Response accuracy analyses for all three CWST types revealed no significant main effects for time and condition or no significant interaction effect.

**Table 2:** Changes in reaction times and response accuracies of the three color–word Stroop tasks throughout ST-LRE and HRE sessions

	Time points						*P* values		
	Baseline	Pre-EX	Post-EX 0	Post-EX 10	Post-EX 20	Post-EX 30	Condition	Time	Interaction
Reaction time (ms)									
Congruent task									
ST-LRE	9285 ± 378	9321 ± 353	8907 ± 356	8955 ± 389	8974 ± 344	9027 ± 326	0.580	0.006	0.999
HRE	9376 ± 404	9393 ± 397	8951 ± 333	9039 ± 379	9057 ± 377	9193 ± 342			
Neutral task									
ST-LRE	9611 ± 354	9702 ± 376	9235 ± 388^b^	9457 ± 333	9400 ± 313	9599 ± 373	0.488	< 0.001	0.805
HRE	9660 ± 355	9539 ± 383	9151 ± 385^a^	9267 ± 376	9414 ± 371	9453 ± 313			
Incongruent task									
ST-LRE	10,614 ± 380	10,708 ± 387	9826 ± 412^ab^	9970 ± 345^ab^	9880 ± 357^ab^	10,173 ± 386	0.529	< 0.001	0.777
HRE	10,668 ± 397	10,532 ± 424	9724 ± 426^ab^	9811 ± 419^ab^	9913 ± 394^ab^	9998 ± 340^a^			
Response accuracy (%)									
Congruent task									
ST-LRE	97 ± 1	96 ± 1	95 ± 1	96 ± 1	97 ± 1	96 ± 1	0.113	0.639	0.650
HRE	96 ± 1	95 ± 1	95 ± 1	95 ± 1	96 ± 1	96 ± 1			
Neutral task									
ST-LRE	96 ± 1	96 ± 1	96 ± 1	97 ± 1	96 ± 1	96 ± 1	0.242	0.347	0.560
HRE	97 ± 1	96 ± 1	95 ± 1	96 ± 1	95 ± 1	95 ± 1			
Incongruent task									
ST-LRE	95 ± 1	95 ± 1	96 ± 1	96 ± 1	96 ± 1	95 ± 1	0.582	0.566	0.153
HRE	96 ± 1	96 ± 1	94 ± 1	95 ± 1	95 ± 1	95 ± 1			

Values are presented as mean ± SEM. ^a^*P* < 0.05 vs. Baseline; ^b^*P* < 0.05 vs. Pre-EX

**Fig. 3:**
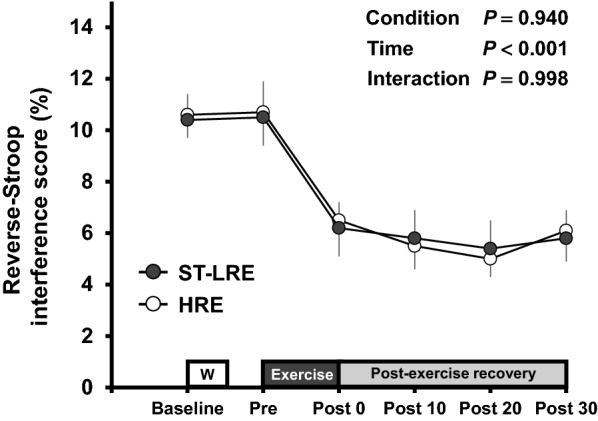
Changes in the reverse-Stroop interference scores throughout ST-LRE and HRE sessions. Values are presented as mean ± SEM. Individual *P* values and effect sizes [95% confidence intervals] among time points are summarized in Table 3

Changes in the reverse-Stroop interference scores throughout ST-LRE and HRE sessions are presented in Fig. 3. The reverse-Stroop interference score analysis revealed a significant main effect for time (*F*_(5,75)_ = 14.91, *P* < 0.001, *η*_p_^2^ = 0.50). The reverse-Stroop interference score significantly decreased after ST-LRE and HRE compared with that before each exercise (see Table 3). Effect sizes for comparison of the reverse-Stroop interference scores before (i.e., baseline and pre-exercise) and after ST-LRE and HRE are listed in Table 3. Large-sized effects were observed between the reverse-Stroop interference scores before and immediately after ST-LRE (*d* = 1.31 and 1.07 vs. baseline and pre-exercise, respectively) and HRE (*d* = 1.14 and 0.96 vs. baseline and pre-exercise, respectively), suggesting that IC largely improved immediately after both protocols. Large-sized effects were also observed between the reverse-Stroop interference scores before and 30 min after ST-LRE (*d* = 1.40 and 1.14 vs. baseline and pre-exercise, respectively) and HRE (*d* = 1.40 and 1.13 vs. baseline and pre-exercise, respectively), suggesting that the improved IC sustained throughout the 30-min post-exercise recovery period for both protocols.

**Table 3:** Effect size and 95% confidence interval (CI) for comparison of the reverse-Stroop interference score between before and after ST-LRE and HRE

	ST-LRE		HRE	
	Baseline	Pre-EX	Baseline	Pre-EX
Post-EX 0 min	1.31 [0.55 to 2.07]	1.06 [0.33 to 1.80]	1.14 [0.40 to 1.89]^*^	0.95 [0.23 to 1.68]^*P* = 0.064^
Post-EX 10 min	1.44 [0.67 to 2.22]^*^	1.21 [0.46 to 1.96]^†^	1.26 [0.50 to 2.01]^*P* = 0.054^	1.05 [0.31 to 1.78]^*P* = 0.065^
Post-EX 20 min	1.84 [1.02 to 2.66]^*^	1.47 [0.69 to 2.25]^†^	1.37 [0.60 to 2.13]^*^	1.14 [0.40 to 1.89]^*P* = 0.071^
Post-EX 30 min	1.40 [0.63 to 2.16]^*^	1.14 [0.40 to 1.89]^†^	1.40 [0.63 to 2.17]^*^	0.13 [0.39 to 1.87]

Values are presented as Cohen’s *d* [95% CI]. Time points at Baseline and Pre-EX were designated “before exercise”. Time points at immediately after exercise (i.e., Post-EX 0 min) and during the 30-min post-exercise recovery period (i.e., Post-EX 10 min, 20 min, 30 min) were designated “after exercise” ^*^Significant difference (*P* < 0.05) between Baseline and post-exercise recovery periods. ^†^Significant difference (*P* < 0.05) between Pre-EX and post-exercise recovery periods

### Changes in psychological conditions for the CWST throughout experimental session

Changes in psychological conditions for the CWST throughout ST-LRE and HRE sessions are shown in Table 4. Arousal analysis revealed a significant main effect for time (*F*_(2.35, 35.17)_ = 38.97, *P* < 0.001, *η*_p_^2^ = 0.72). Arousal significantly increased immediately after ST-LRE and HRE compared with that before each exercise (all *P*s < 0.001, *d* = 2.22 to 3.03 vs. baseline and pre-exercise for both protocols), and the increased arousal remained significant until the 10-min post-exercise recovery period for both protocols (all *P*s < 0.05, *d* = 0.98 to 1.64 vs. baseline and pre-exercise for both protocols). Analyses of mental fatigue and motivation revealed significant effects for time (*F*_(2.26, 33.96)_ = 45.30, *P* < 0.001, *η*_p_^2^ = 0.75 and *F*_(2.71, 40.73)_ = 3.40, *P* = 0.030, *η*_p_^2^ = 0.19, respectively). A trend toward significance was obtained in main effects for condition and time of ability to concentration (*F*_(1,15)_ = 3.64, *P* = 0.076, *η*_p_^2^ = 0.20 and *F*_(2.30, 34.46)_ = 3.04, *P* = 0.054, *η*_p_^2^ = 0.17, respectively). Mental fatigue significantly increased immediately after ST-LRE and HRE compared with that before each exercise (all *P*s < 0.001, *d* = 2.35 to 3.77 vs. baseline and pre-exercise for both protocols), and the increased mental fatigue remained significant until the 30-min post-exercise recovery period for both protocols (all *P*s < 0.01, *d* = 0.93 to 1.45 vs. baseline and pre-exercise for both protocols). Motivation significantly increased immediately after only a ST-LRE (*P* = 0.048, *d* = 0.76 vs. pre-exercise).

**Table 4:** Psychological conditions for the color–word Stroop task throughout ST-LRE and HRE sessions

	Time points						*P* values		
	Baseline	Pre-EX	Post-EX 0	Post-EX 10	Post-EX 20	Post-EX 30	Condition	Time	Interaction
Arousal									
ST-LRE	2.6 ± 0.2	3.0 ± 0.2	4.8 ± 0.2^ab^	3.9 ± 0.2^abc^	3.2 ± 0.2^c^	3.0 ± 0.2^c^	0.446	< 0.001	0.687
HRE	2.6 ± 0.2	2.9 ± 0.2	4.8 ± 0.2^ab^	3.6 ± 0.2^abc^	3.1 ± 0.2cd	2.9 ± 0.2cd			
Mental fatigue (mm)									
ST-LRE	19.9 ± 4.7	23.4 ± 5.6	69.8 ± 4.2^ab^	61.2 ± 3.8^ab^	53.9 ± 5.2^ab^	51.8 ± 6.2^ab^	0.114	< 0.001	0.283
HRE	16.8 ± 5.3	17.2 ± 4.5	69.8 ± 2.0^ab^	52.7 ± 4.8^abc^	49.8 ± 6.0^abc^	40.1 ± 7.1^abce^			
Concentration (mm)									
ST-LRE	69.4 ± 3.5	61.8 ± 4.2	76.5 ± 4.2	65.9 ± 3.8	62.1 ± 4.4	65.6 ± 4.0	0.076	0.054	0.571
HRE	58.9 ± 5.7	56.6 ± 4.7	67.6 ± 4.7	65.3 ± 3.5	60.6 ± 4.1	59.3 ± 4.3			
Motivation (mm)									
ST-LRE	67.8 ± 4.8	64.1 ± 4.0	76.4 ± 4.1^b^	71.6 ± 3.4	64.3 ± 5.0	69.1 ± 4.4	0.644	0.030	0.682
HRE	69.9 ± 3.9	64.8 ± 3.4	72.7 ± 4.2	69.3 ± 4.0	64.6 ± 4.7	66.4 ± 4.1			

Values are presented as mean ± SEM. ^a^*P* < 0.05 vs. Baseline; ^b^*P* < 0.05 vs. Pre-EX; ^c^*P* < 0.05 vs. Post-EX 0 min; ^d^*P* < 0.05 vs. Post-EX 10 min; ^e^*P* < 0.05 vs. Post-EX 20 min

## Discussion

Our previous study found that immediate post-exercise improvement in IC was greater with HRE than with LRE [17]. In contrast, this study obtained that immediate post-exercise IC improvement did not differ between ST-LRE and HRE. Furthermore, the degree of post-exercise IC improvements throughout the 30-min post-exercise recovery was similar between the two protocols. These findings suggest that ST-LRE improves post-exercise IC in a manner similar to that by HRE.

Our previous study found that although the degree (i.e., immediate effect) of improvement in IC immediately after aerobic exercise did not differ between high-intensity interval exercise and moderate-intensity continuous exercise, the duration (i.e., sustainable effect) of significant improvements in IC throughout the 30-min post-exercise recovery period was longer following high-intensity interval exercise than following moderate-intensity continuous exercise [36]. Thus, in addition to the immediate effect, the sustainable effect following exercise may be an important factor for understanding the potential effect on post-exercise IC improvements among exercise protocols [16, 32–37]. Several studies have examined the sustainable effects of resistance exercise on post-exercise IC improvements [13, 14, 16]. Of those, Johnson et al. [14] reported that although IC improved immediately after both moderate-intensity whole-body resistance exercises (i.e., 60% 1-RM) with 10-min and 30-min durations, the IC improvements reversed at the 30-min post-exercise recovery period. Their findings suggest that duration of IC improvements following resistance exercise may be less than 30 min. Nevertheless, the study by Johnson et al. [14] recruited older individuals, and therefore, the sustainable effect of post-exercise IC improvements may be limited in this population. In a recent study, we identified that post-exercise IC improvements induced by knee extensor HRE (i.e., 70% 1-RM, 10 repetitions/set, and 6 sets) and high-volume LRE (i.e., 35% 1-RM, 20 repetitions/set, and 6 sets) were sustained until 20 min after both protocols [16]. Furthermore, in the present study, we observed that post-exercise IC improvements induced by knee extensor ST-LRE and HRE were sustained until 30 min after both protocols. Therefore, both ST-LRE and HRE may potentially have a sustainable effect on post-exercise IC improvements for up to 30 min after exercise in healthy young adults.

The circulating lactate is utilized as an important energy substrate during exercise in the human brain [25]. Our previous studies demonstrated that aerobic exercise-induced IC improvements are related to circulating lactate levels [28, 30], potentially by increasing cerebral lactate metabolism [28]. In the present study, we obtained that resistance exercise-induced increase in blood lactate levels did not differ between ST-LRE and HRE. This present finding corroborates the results of previous study [19, 20]. Therefore, the findings of the present and previous studies may contribute to our understanding of similar post-exercise IC improvements between ST-LRE and HRE.

We and others have previously reported that psychological and perceptual responses induced by exercise may be associated with post-exercise IC improvements [16, 17, 26, 31–33, 37]. Byun et al. [26] reported that an exercise-induced increase in arousal is correlated with improved cognitive function and increased cerebral neural activity. Moreover, we reported that the level of increase in RPE during exercise may be associated with the degree of post-exercise IC improvements [16, 17, 31, 37] and may potentially represent increased cerebral neural activity [46]. In the present study, the levels of the increases in arousal and RPE induced by resistance exercise did not differ between ST-LRE and HRE. Additionally, the present study observed similar increase in mental fatigue between the two protocols. Grego et al. [47] reported that aerobic exercise of a very long duration (i.e., 3 h) decreased cognitive function, which may be due to increased mental fatigue. However, our previous studies have shown that the degrees of IC improvements after both aerobic and resistance exercises were partially in accordance with the levels of increased mental fatigue [16, 17, 32, 33, 37]. The durations of the exercise protocols used in our studies were within the reasonable ranges based on general exercise guidelines [5, 6, 48, 49]. Hence, when an exercise protocol is performed within a reasonable duration, the increased mental fatigue may play an important role in improving post-exercise IC, because it may possibly represent increased cerebral neural activity. Therefore, the similar responses for psychological and perceptual parameters between ST-LRE and HRE may also contribute to our understanding of their equivalent effects on post-exercise IC improvements.

The magnitude of muscle activation during resistance exercise may be related to the degree of cerebral neural activation [50, 51]. In this study, peak iEMG activities of the three quadriceps femoris muscles during resistance exercise were higher for HRE than for ST-LRE. This suggests that although ST-LRE may contribute to preventing injuries to the musculoskeletal system, the lower EMG activity during ST-LRE may indicate weaker effects on increasing cerebral neural activity and improving cognitive function compared to HRE. Nevertheless, the quadriceps femoris EMG activation during ST-LRE is more continuous than that during HRE, because ST-LRE is performed with slow contractile actions during the concentric and eccentric phases and isometric actions during the rest phase between repetitions. Considering this muscle contractile behavior during ST-LRE, continued duration and peak magnitude of muscle activation during resistance exercise may contribute to increasing cerebral neural activity and improving post-exercise IC. Indeed, the continued EMG activation of the quadriceps femoris during ST-LRE may result in a blood lactate response similarly to those during HRE, potentially due to increased metabolic stress in the exercising muscles [38]. This may corroborate the result of our previous study that the blood lactate response induced by high-volume LRE (i.e., increased duration of muscle activity) was similar to that induced by HRE [16]. Therefore, longer neural activity of the exercising muscles during ST-LRE than during HRE may counter the weakness of ST-LRE regarding the relationship between peak muscle activation and post-exercise IC improvements.

This study observed that mean value of SBP, but not DBP, during exercise was lower for ST-LRE than for HRE in healthy young adults. This finding corroborates the results of studies by Tanimoto et al. [19, 20] who recruited healthy young adults. Therefore, compared to HRE, ST-LRE may be useful in mitigating blood pressure response during resistance exercise, with the similar improvements in post-exercise IC, at least in healthy young individuals.

This study recruited healthy young adults in a preliminary safety study in order to identify whether ST-LRE would be an effective protocol to improve cognitive function in various populations. However, this is a major limitation of this study, because effective resistance exercise protocols to improve cognitive function are generally more important for older individuals and patients with chronic diseases than for healthy young individuals. Previous studies have reported that long-term ST-LRE increases muscle size and strength effectively in older individuals and patients with chronic diseases [21–23]. Therefore, to achieve our goal, further studies are needed to determine the effects of ST-LRE on post-exercise IC improvements in these populations.

In conclusion, this study found that the degree of post-exercise IC improvements was similar between ST-LRE and HRE in healthy young adults. Thus, in the present preliminary study, we suggest that despite the application of a lower exercise load, ST-LRE improves post-exercise IC similarly to HRE in this young population.

## Data Availability

Data will be provided by the corresponding author upon request. This study was approved by the Ethics Committee of Ritsumeikan University and conducted according to the Declaration of Helsinki. Informed written consent was obtained from all participants.

## Abbreviations

1-RM: One-repetition maximum
CWST: Color–word Stroop task
DBP: Diastolic blood pressure
EMG: Electromyography
FAS: Felt Arousal Scale
HR: Heart rate
HRE: High-intensity resistance exercise
IC: Inhibitory control
LRE: Low-intensity resistance exercise
SBP: Systolic blood pressure
ST: Slow movement and tonic force generation
VAS: Visual Analogue Scale
*η*_p_^2^: Partial eta-squared
